# Development of a cardiac loading device to monitor cardiac function during *ex vivo* graft perfusion

**DOI:** 10.1371/journal.pone.0195721

**Published:** 2018-04-27

**Authors:** Emilie Farine, Manuel U. Egle, Alice C. Boone, Sandro Christensen, Thierry P. Carrel, Hendrik T. Tevaearai Stahel, Sarah L. Longnus

**Affiliations:** Department of Cardiovascular Surgery, Inselspital, Bern University Hospital, Bern, Switzerland; Scuola Superiore Sant'Anna, ITALY

## Abstract

**Background:**

*Ex vivo* heart perfusion systems, allowing continuous perfusion of the coronary vasculature, have recently been introduced to limit ischemic time of donor hearts prior to transplantation. Hearts are, however, perfused in an unloaded manner (via the aorta) and therefore, cardiac contractile function cannot be reliably evaluated.

**Objectives:**

We aim to develop a ventricular loading device that enables monitoring of myocardial function in an *ex vivo* perfusion system. In this initial study, was to develop a prototype for rat experimentation.

**Methods:**

We designed a device consisting of a ventricular balloon and a reservoir balloon, connected through an electronic check valve, which opens and closes in coordination with changes in ventricular pressure. All balloons were produced in our laboratory and their properties, particularly pressure-volume relationships, were characterized. We developed a mock ventricle *in vitro* test system to evaluate the device, which was ultimately tested in *ex vivo* perfused rat hearts.

**Results:**

Balloon production was consistent and balloon properties were maintained over time and with use on the device. Results from *in vitro* and *ex vivo* experiments show that the device functions appropriately; hemodynamic function can be measured and compares well to measurements made in an isolated, working (loaded) rat heart preparation.

**Conclusions:**

Our cardiac loading device appears to reliably allow measurement of several left ventricular hemodynamic parameters and provides the opportunity to control ventricular load.

## Introduction

Heart transplantation (HTx) remains the gold standard treatment to improve quality of life and survival in patients with advanced heart failure. Despite acceptable results, improvement is still possible and urgently needed. Indeed, although survival after HTx has remarkably improved over the last 2 decades[1], the incidence and mortality from primary graft dysfunction (PGD) remains elevated[2,3] contributing to up to 30% of post-transplantation deaths. Furthermore, insufficient supply of grafts has become critical. Increasing the number of potentially available organs with marginal donors and donation after circulatory death (DCD), in addition to conventional donation after brain death (DBD), are promising options. However, with marginal donation and DCD, evaluation of graft suitability for transplantation takes on an even greater importance. For example, DCD organs undergo an inevitable period of warm, global ischemia, which requires specialized strategies to limit ischemia-reperfusion injury and ensure adequate post-transplant functional recovery[4].

Recently, an *ex vivo* cardiac graft perfusion system, the Organ Care System (OCS) from TransMedics™[5], has become available. The OCS was first developed and tested in clinical practice for DBD HTx, showing intermediate outcomes similar to standard static storage[6], thereby attesting to its safety. It has also been introduced for DCD HTx to protect cardiac grafts from additional ischemia and to monitor cardiac function. The current system allows continuous perfusion of the coronary vasculature (via the aorta) during graft storage and transport. This however is performed in a Langendorff / unloaded mode i.e. the heart returns to a beating state, but the left ventricle (LV) remains unloaded. In this configuration, cardiac function cannot be fully evaluated.

In recent reports of DCD HTx, two differing approaches have been used towards graft evaluation. In the first, cardiac grafts are returned to the beating state while perfused on the OCS, during which time the graft evaluated by several parameters including lactate release/ extraction [7]. The second approach involves *in vivo* normothermic regional perfusion (NRP)[8]. For this, the heart is reperfused *in vivo* with extracorporeal membrane oxygenation (ECMO) after declaration of death. The heart is then weaned from ECMO and measurement of hemodynamic function is possible via catheterization and echocardiography. Interestingly, graft evaluations using arterial lactate profile measured on the OCS are not always concordant with *in vivo* functional assessment. This discrepancy clearly demonstrates the importance of improving methods to evaluate cardiac graft function with DCD.

Since *ex vivo* cardiac graft perfusion is becoming established in heart transplantation, it is highly desirable to identify methods for a more thorough assessment of contractile function, without requiring *in vivo* heart evaluation or complex, *ex vivo*, working mode graft perfusions.

Our ultimate objective is to develop a loading device that can be adapted to currently used perfusion systems such as the OCS and that would allow a precise and reliable evaluation of the cardiac graft contractile function. In the current study, we aimed to develop a miniaturized device for rat experimentation and to verify its ability to monitor cardiac hemodynamic function using an *ex vivo* perfused rat heart model.

## Methods

### Device description

**Fig 1:**
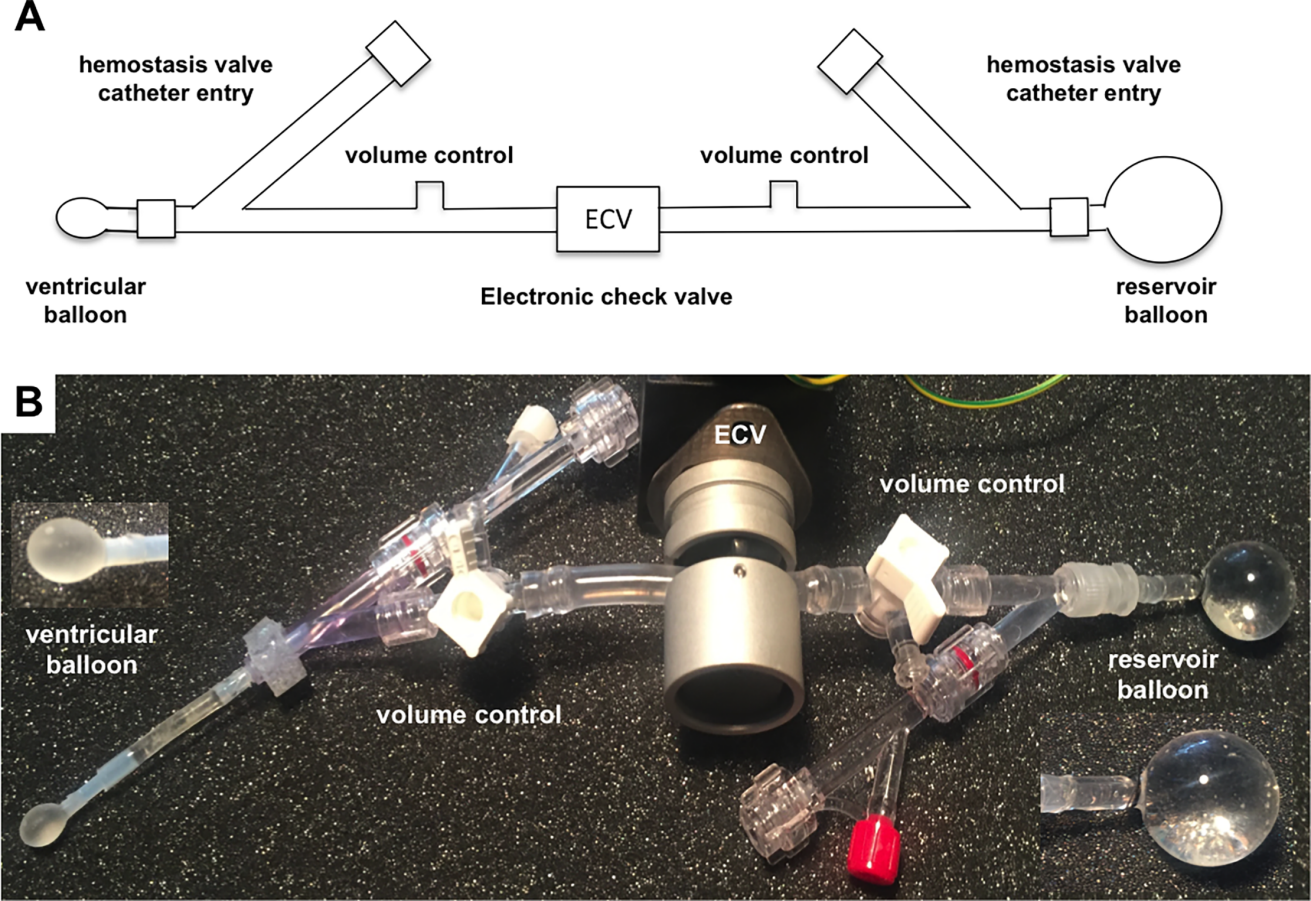
Design of cardiac loading device. (A) The cardiac loading device (CLD) consists of a ventricle balloon and a reservoir balloon connected by tubing, in which an electronic check valve (ECV) is placed. In each section of tubing between the ECV and balloon, a hemostasis valve and a syringe port are located for insertion of catheters and for control of device volume (pressure), respectively; (B) Photo of the CLD.

We designed a cardiac loading device (CLD) consisting of a ventricular balloon (VB, with a volume of either 240 or 280 μL) and a reservoir balloon (RB, with a volume of 4.2 mL), connected with tubing and filled with water. An electronic check valve (ECV; 2/2 NC solenoid pinch valve series 284, ASCO Numatics, Florham Park, NJ, USA) controls the flow between the balloons in a binary manner, as shown in Fig 1. With this design, the water can move freely between the balloons through the ECV when it is open, but the volumes in each section of the device (ventricular or reservoir) remain fixed when the ECV is closed. One 3-way stopcock and one hemostasis valve are placed on either side of the ECV to permit, respectively, volume adjustment and insertion of micro-tip pressure catheters (Millar, Houston, USA). The pressure catheters are used for continuous measurement of internal balloon pressures.

**Fig 2:**
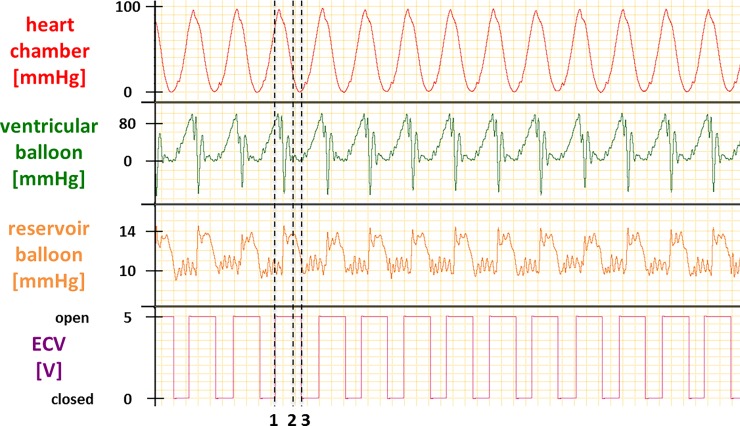
*In vitro* ventricular simulator tests. *In vitro* ventricular simulator testing demonstrates appropriate functioning of balloon device: **1:** pressure in heart chamber increases, causing pressure in VB to exceed set afterload and opening if ECV; this is followed by VB emptying (pressure decreases) and RB filling (pressure increases) **2:** pressure in VB falls below pressure in RB, this results in refilling of VB **3:** refilling of VB complete; pressure in RB falls below preload pressure; ECV closes.

The CLD was designed for use with the isolated, perfused rat heart preparation. The VB is inserted into the LV via the mitral valve, while the RB remains outside the heart. The internal balloon pressures are used to control the ECV. When the pressure in the VB exceeds a chosen (pre-set) ‘afterload’ value, the ECV opens. The ECV is set to close after a chosen (pre-set) delay period. As such, ECV function corresponds with the cardiac cycle as follows: ECV opening occurs at the time of aortic valve opening, and ECV closing occurs at the time of mitral valve closing. The function of the device over the cardiac cycle is detailed in Fig 2. Pressures are recorded with a PowerLab data acquisition system (ADInstruments, Spechbach, Germany) and the ECV is controlled using the ‘Fast Response Output’ add-on of LabChart (ADInstruments, Spechbach, Germany).

### Balloon production

All balloons were designed and produced in our laboratory. Two LV ellipsoid Plexiglas positives for VBs were designed with volumes of 240 or 280 μL, estimated using previously obtained data[4]. RBs were designed with a spherical shape and a larger volume of 4.2 mL, to limit pressure in the device with the maximal volume increase of 280 μL (occurs only if VB empties completely).

VBs were custom-made using Plexiglas positives and AlpaSil 32 Silicone A + B (Silitec AG; Gümligen, Switzerland). The silicone mixture was de-gassed by centrifugation at 4500G for 1 minute at 18°C. The balloon positives were then immediately dipped in de-gassed silicone and centrifuged at 44G for 5 minutes at 18°C. Balloons were polymerized at room temperature for 24 hours, and then removed by gently sliding them off.

Because of the much larger size, RBs were manufactured using a slightly different protocol. First, a negative mold was produced using Bluesil RTV silicone (Silitec AG; Gümligen, Switzerland) and a Plexiglas positive. The silicone mixture was de-gassed by centrifugation (as above) and the positive was placed in the RTV silicone. After 24 hours of polymerization, the RTV silicone mold was cut to remove the Plexiglas positive. The RTV silicone mold was then used to produce positives with water-soluble wax (Freeman Sol-U-Carv wax, Freeman Manufacturing & Supply Company, Avon, USA). To do so, the wax was melted by heating and injected into the bottom of the mold. After 20 minutes, the solidified wax positive was gently removed from the molds, dipped in de-gassed AlpaSil 32 Silicone mixture, and centrifuged at 44G for 5 minutes at 18°C. Balloons were then polymerized for 24 hours at room temperature and wax removed by solubilization in hot water.

### Balloon characterization

All balloons were characterized by thickness, measured at room temperature (RT) at the neck using digital electronic calipers (Fine Science Tools FST, Heidelberg, Germany), and by pressure-volume (PV) relationships.

PV relationship tests were repeated at RT to determine the stability of the balloon characteristics over time ('weekly' tests) and with use. For this, a test system was established by connecting the balloon with a pressure transducer (TruWave Disposable Pressure Transducer, Edwards Lifesciences; Irvine, California, USA) via a 3-way stopcock. The 3-way stopcock was also connected to a 5 mL syringe and a 250 μL Hamilton syringe for volume adjustment and measurement. The whole system was filled with water and completely de-gassed. Pressure within the system was continuously recorded using a PowerLab data acquisition system.

For VBs, PV tests were performed over the expected working volume range and beyond. Briefly, the VB was filled to the maximum filling volume (i.e. 240/280 μL), and then volume in the balloon was increased by 50 μL (increments of 10 μL) using a Hamilton syringe and then returned to its initial value. The volume was then decreased, with increments of 10 μL, by 240/280 μL (maximum filling volume). The maximal working volume was defined as the maximum volume at which the balloon did not exert any pressure.

The procedure was slightly different for RBs. After setting the initial volume to a pressure of 11.5 mmHg (standard preload pressure), we first decreased the volume in the balloon by 50 μL, returned to initial volume, and then increased the volume by 250 μL (always with increments of 10 μL). The elastic resistance of the reservoir balloons, also known as the elastance, was then determined by calculating the slope of the PV curve by linear regression[9], as follows:

$$slope=\frac{dP}{dV}=elastance$$

For VBs and RBs, PV relation measurements were repeatedly performed (according to the procedure described above) once a week over 24 weeks to detect possible changes in balloon properties over time.

### *In vitro* ventricle simulator tests

We have also developed an *in vitro* test system to evaluate the function of the CLD. It consists of a pressure generator for the ventricle simulator, connected to a heart chamber. A rotating motor, connected to a power supply, moves a base plate on which the plunger(s) of syringe(s) is (are) fixed. The pressure developed and heart rate can be varied depending on the number of syringes used and the rate at which the base plate moves (maximum of 230 bpm, technical limit), respectively. With this system, we tested the CLD for 1h, at a heart rate of 230 bpm and a developed pressure of 100 mmHg.

### Over use tests

Tests were performed to determine whether the balloon characteristics change with use on the *in vitro* ventricle simulator. First, we measured balloon PV relationships and then used them in the simulator for 1 hour as described above. Thereafter, the PV relationships were measured once again.

### Isolated perfused rat heart tests

#### Ethics statement

All experimental procedures were performed in compliance with the European Convention for Animal Care and approved by the Swiss animal welfare authorities and state veterinary office (Ethics Committee for Animal Experimentation, Bern, Switzerland; Authorization of Animal Experimentation BE88/16). Surgery was performed under anesthesia and all efforts were made to minimize animal suffering.

#### Isolated heart preparation

Adult male Wistar rats (n = 6; 387 ± 17 g; Janvier Labs, Le Genest-Saint-Isle, France), housed under standard conditions with unlimited access to food (standard laboratory diet *ad libitum*) and water, were anesthetized with an intraperitoneal injection of 100 mg/kg ketamine (Narketan™, Vetoquinol AG, Bern, Switzerland) and 10 mg/kg xylazine (Xylapan™, Vetoquinol AG, Bern, Switzerland).

After anesthesia and as soon as the pedal reflex had disappeared, hearts were excised and immediately placed in ice-cold PBS. Hearts were then perfused in an unloaded manner, via the cannulated aorta, with a modified Krebs-Henseleit bicarbonate (KHB) buffer containing: 118 mM NaCl, 4.7 mM KCl, 1.2 mM KH_2_PO_4_, 1.2 mM MgSO_4_ ∙ 7H_2_O, 1.25 mM CaCl_2_ ∙ 7H_2_O, 25 mM NaHCO_3_ and 11 mM glucose[4,10,11], at a constant pressure of 60 mmHg[4,10,11].

Once the left atrium was cannulated, hearts were switched to working (loaded) mode perfusion and a micro-tip pressure catheter (Millar, Houston, USA) was inserted into the LV. The preload was maintained at 11.5 mmHg, and the afterload was kept at 60 mmHg for 20 min and then increased to 80 mmHg for another 20 min. During this time, continuous catheter-based measurements were recorded in the working (loaded) mode for comparison with values obtained using the CLD.

For testing the CLD, hearts were returned to the unloaded mode. The deflated VB was inserted into the LV via the mitral valve. Thereafter, the VB was filled with ddH_2_O using a 250 μL Hamilton syringe, and the CLD was started at a preload of 11.5 mmHg and an afterload of 60 mmHg. The afterload was then increased after 20 min to 80 mmHg for an additional 20 min.

### Data analysis

For PV relationship tests, pressure measurements were first normalized to the initial pressure to limit technical variation.

Unless stated otherwise, values are reported as mean ± SD. Data analysis was performed with GraphPad Prism (version 7.0, GraphPad Software Inc., La Jolla, CA, USA). Differences in the balloon characteristics among batches were evaluated non-parametrically using Mann-Whitney tests. Pairwise comparisons were performed non-parametrically to compare the balloon characteristics over time and over use using Wilcoxon matched-pairs signed rank tests.

All p values were two sided, adjusted for multiple comparisons (modified sequential rejective Bonferroni procedure[12]) and reported after correction. Corrected p values were considered statistically significant if p<0.05.

## Results

A total of 96 balloons were produced (33 RBs and 63 VBs) in thirteen batches (groups of balloons produced together), permitting the evaluation of both production consistency and evaluate balloon properties. We were able to reach a production yield of up to 100% and 60% for the VB and RB, respectively.

### Balloon characterization

#### Baseline measurements

**Fig 3:**
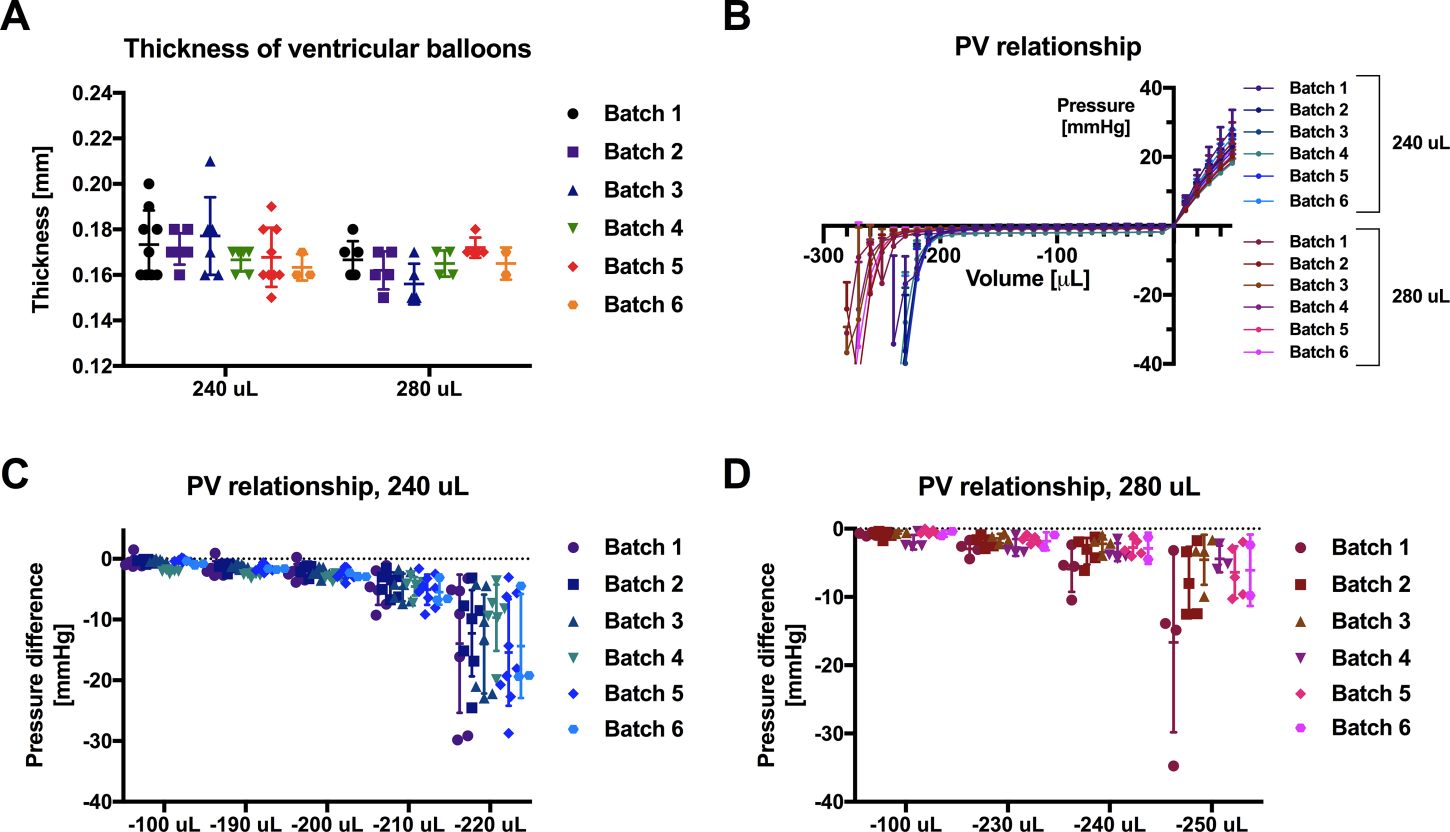
Baseline characteristics of ventricular balloons. (A) Thickness of ventricular balloons (VB); (B) PV relationship of VBs according to batch; (C) pressure for all 240 μL VBs at volumes of -100, -190, -200, -210, -220 μL; (D) pressure for all 280 μL VBs at volumes of -100, -230, -240, -250 μL. Data are normalized to initial pressure (volume = 0) and expressed as mean ± SD. n = 2–9 per group.

All balloons were tested after fabrication. No significant difference in VB thickness or PV relationship was observed between batches (Fig 3A and 3B). We also measured maximal working volumes of 200 μL and 230 μL for the 240 μL and 280 μL VB molds, respectively, as shown in Fig 3C and 3D. When approaching withdrawal of the maximum working volume, pressure changes were observed and variability among balloons dramatically increased.

**Fig 4:**
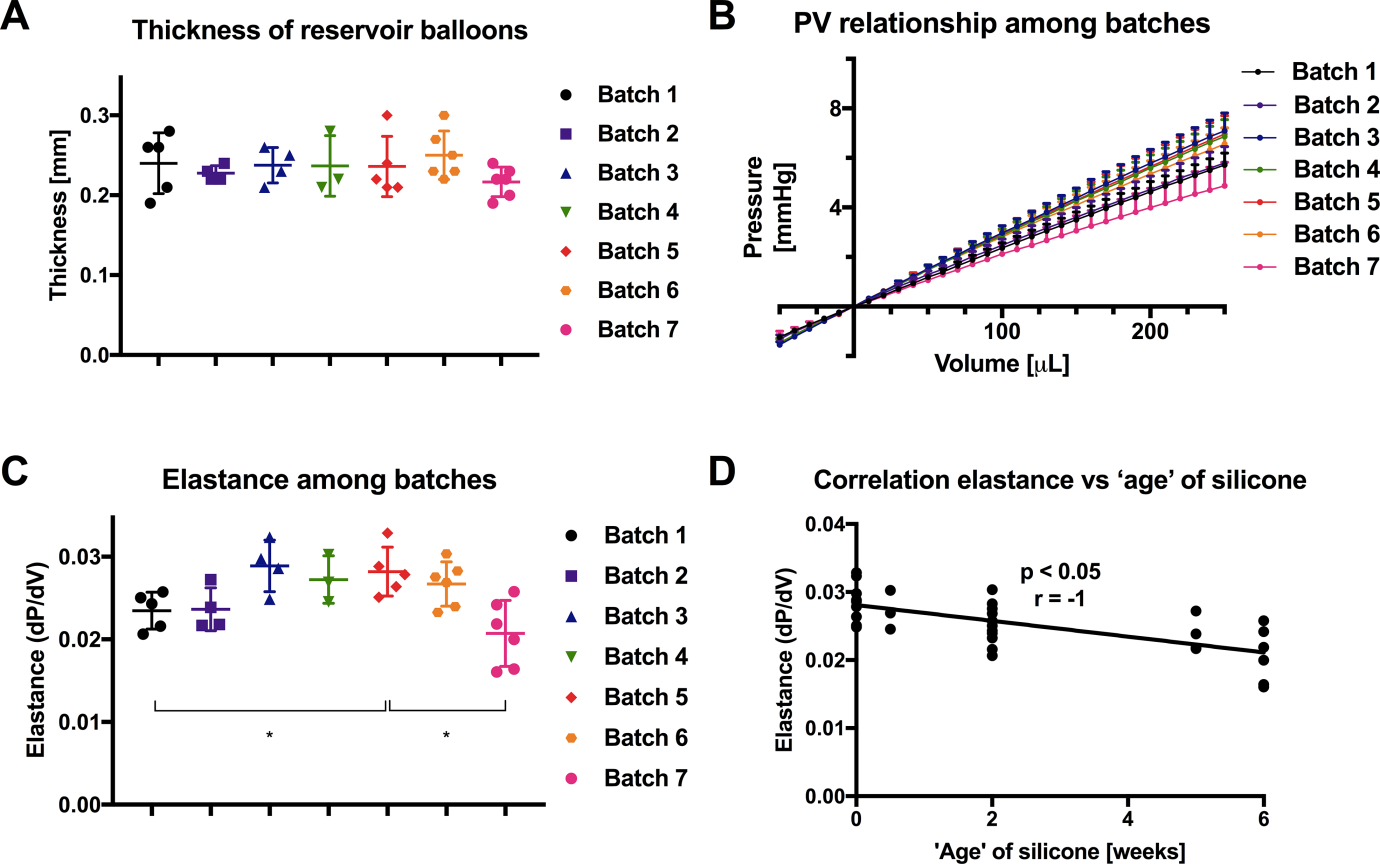
Baseline characteristics of reservoir balloons. (A) Thickness of reservoir balloons (RB); (B) PV relationship of RB according to batch; (C) elastance of RB; (D) negative correlation between the elastance of the RB and silicone ‘age’. Data are normalized to initial pressure (volume = 0) and expressed as mean ± SD. * p<0.05. n = 3–6 per group.

Balloon thickness was also similar between batches of RBs (Fig 4A). However, small differences in the PV relationship and elastance (coefficient of determination R^2^ >0.997) were observed for these balloons (Fig 4B and 4C). A negative correlation (p<0.05) between the elastance of the RB and the ‘age’ of the silicone (i.e. time between the opening of the silicone bottles and the balloon production) was measured, as shown in Fig 4D. For simplicity, elastance, not PV relationships, is reported for reservoir balloon evaluations.

#### Weekly measurements

**Fig 5:**
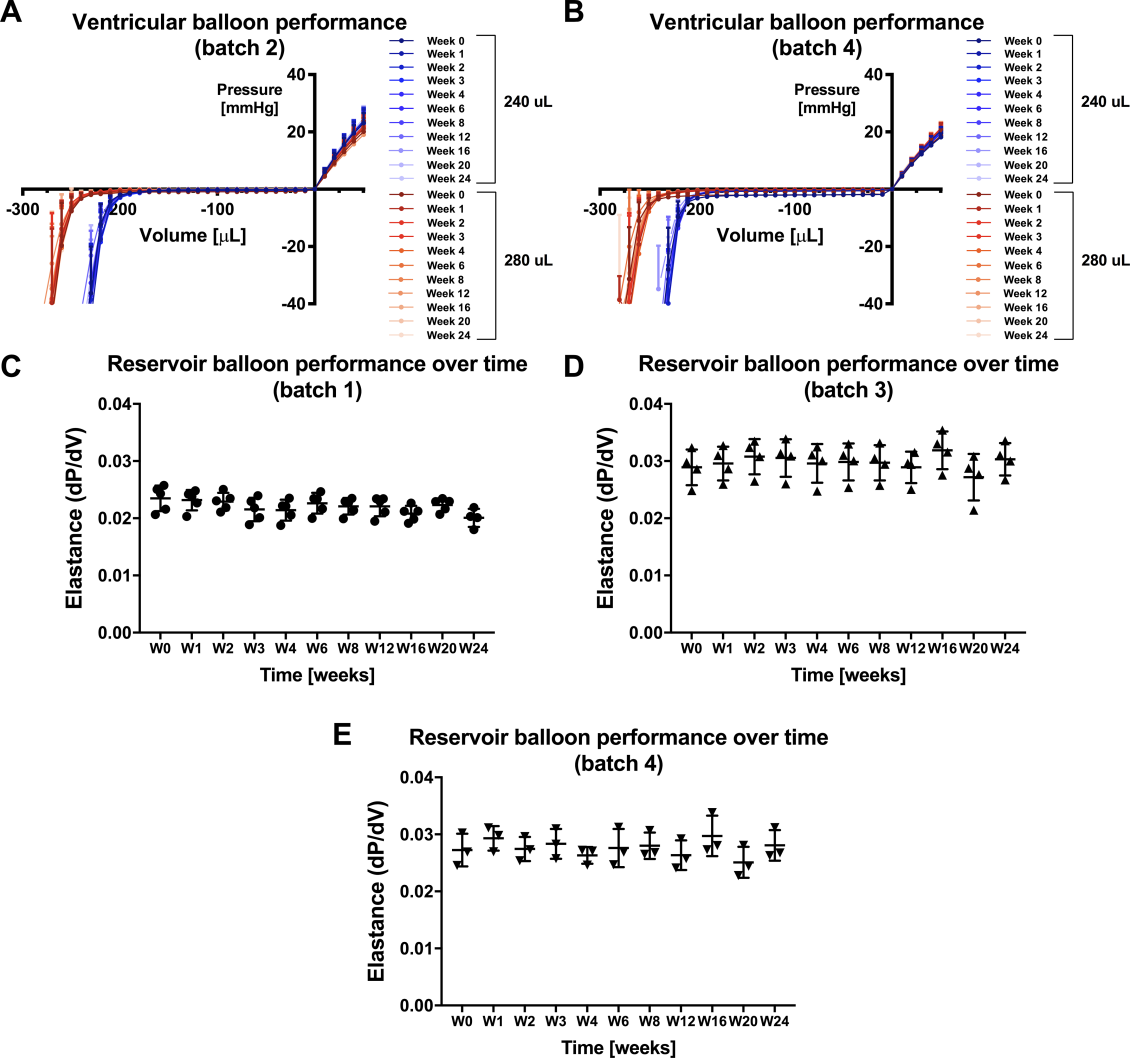
Pressure-volume (PV) relationship for ventricular and reservoir balloons: Weekly tests. Weekly measurements of PV relationships for VB batches 2 (A) and 4 (B); Reservoir balloon elastance for batches 1 (C), 3 (D) and 4 (E). Week 0 and 1 correspond to testing within one week of production and one week after production, respectively. Data are normalized to initial pressure (volume = 0) and expressed as mean ± SD. n = 3–7 per group.

No change in PV relationships or maximal working volume of the VB (batches 2 & 4) was measured over time for up to 24 weeks after fabrication (Fig 5A and 5B). Similarly, no change in elastance of the RB (batches 1, 3 & 4) was observed over time for up to 24 weeks after production (Fig 5C–5E).

#### *In vitro* ventricle simulator tests

An example of an *in vitro* test is presented in Fig 2. The pressure in the VB increases according to the pressure generated in the ventricle simulator. When the VB pressure exceeds the set afterload (e.g. 80 mmHg in an example of curves presented in Fig 2), the ECV opens (Fig 2, Line 1). The VB pressure briefly overshoots the afterload pressure, then it falls (VB empties), and the RB expands (pressure increases). When VB pressure falls below RB pressure, refilling of the VB starts (Fig 2, Line 2). Finally, when VB refilling is complete, the pressure in the RB falls below the preload pressure (e.g. 11.5 mmHg) and the ECV closes (Fig 2, Line 3). This completes one heart cycle.

Using the ventricle simulator, we have determined the optimal valve delay time (t) for several heart rates (x; 125–230 bpm), fitting it to a polynomial curve (R^2^ = 0.983):

$$t=f(x)=-1.574\;10^{-8}\;*\;x^{3}+1.056\;10^{-5}*\;x^{2}-3.028\;10^{-3}*\;x+0.5155$$

#### Over use measurements

**Fig 6:**
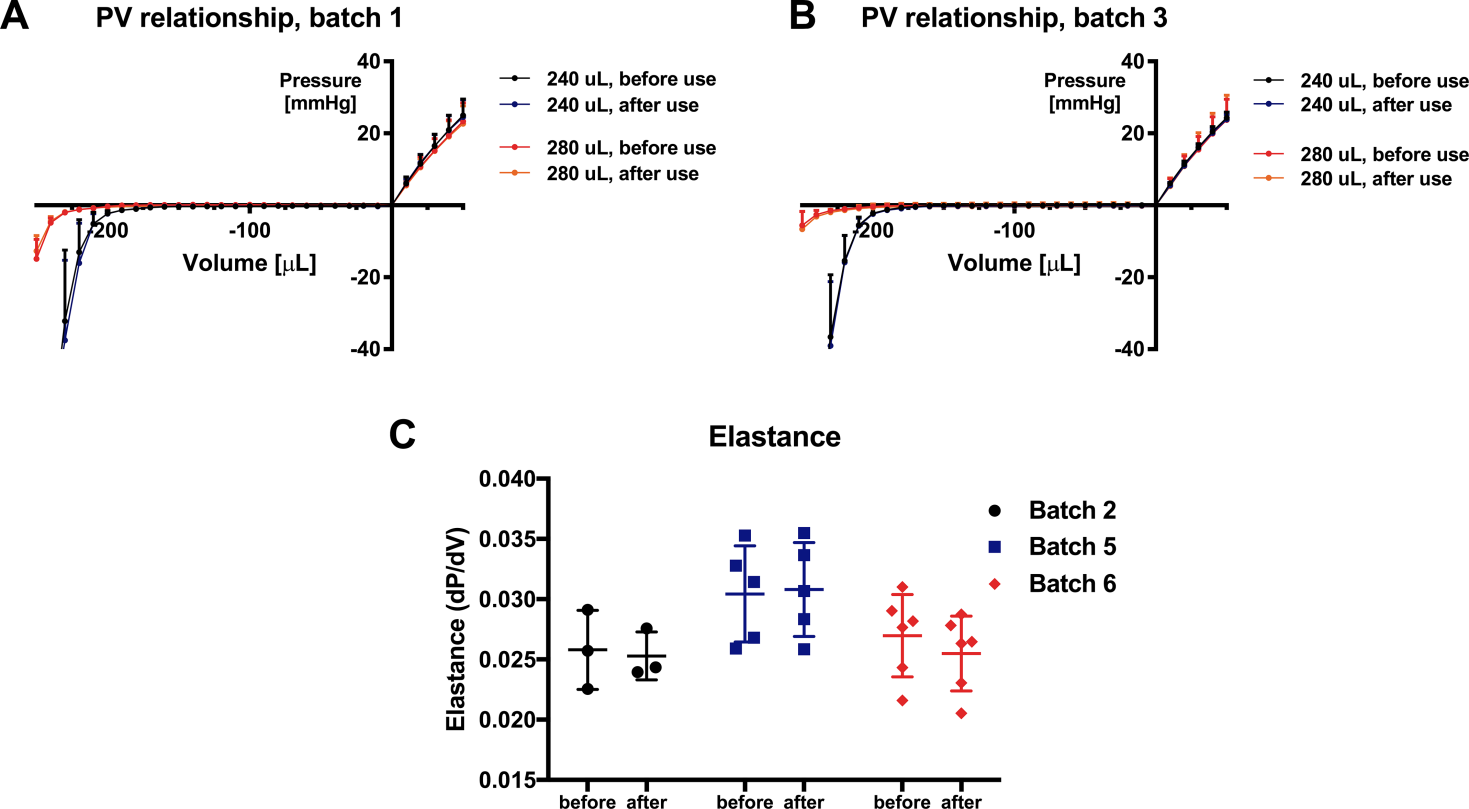
Pressure-volume (PV) relationships for ventricular and reservoir balloons: With use tests. PV relationships (VB) for batches 1 (A) and 3 (B) and calculated elastance (RB) for batches 2, 5 and 6 (C) measured before and after one-hour use on ventricular simulator. Data are normalized to initial pressure (volume = 0) and expressed as mean ± SD. n = 3–6 per group.

We did not note any difference in PV relationship, nor in maximal working volume of VBs (batches 1 & 3) after use on the CLD for one hour (Fig 6A and 6B). Similarly, no difference in elastance of RBs (batches 2 & 5) was observed (Fig 6C).

### Isolated perfused rat heart tests

**Fig 7:**
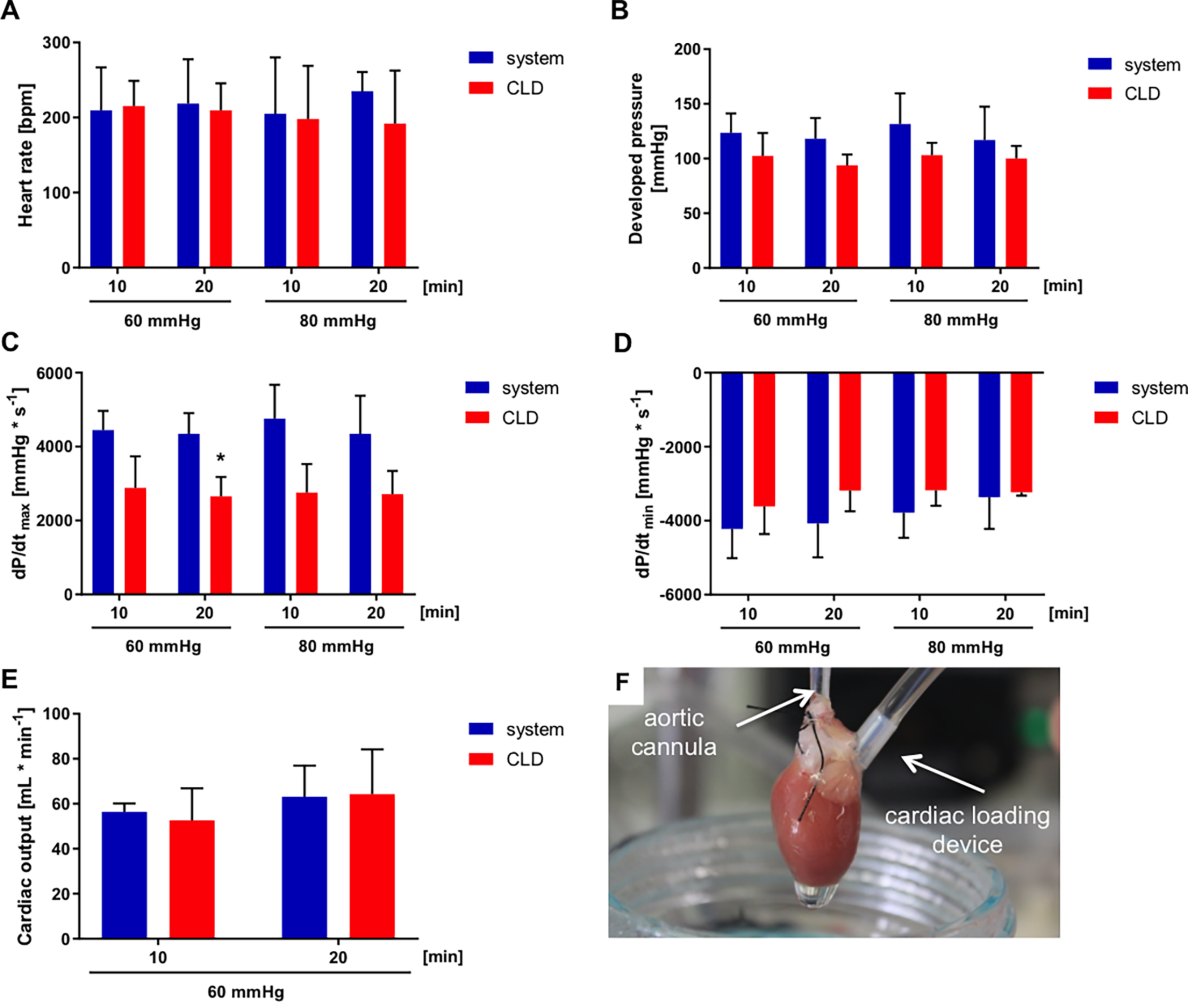
Results of isolated rat hearts. Heart rate (A), developed pressure (B), dP/dt_max_ (C), dP/dt_min_ (D), cardiac output (E) with the isolated working rat heart system (system) and our cardiac loading device (CLD), for afterloads of 60 and 80 mmHg. (F) Picture of CLD in an isolated rat heart. Data are expressed as mean ± SD. * p<0.05, n = 3–6 per group.

Our CLD allows the assessment of heart rate, developed pressure, dP/dt_max_ and dP/dt_min_ in isolated beating rat hearts. Overall, no significant difference (p>0.05) was observed when compared to measurements obtained with the working (loaded) heart system at 60 mmHg, except for dP/dt_max_ at 20 minutes as shown in Fig 7. However, the CLD tended to underestimate the values of developed pressure (correction factor: 0.88±0.23), dP/dt_max_ (0.63±0.18), dP/dt_min_ (0.90±0.21), and overestimate cardiac output (1.13±0.38).

## Discussion

We present initial results with a cardiac loading device conceptualized and developed for the functional evaluation of isolated hearts. This device permits left ventricle loading and ejection, and therefore, measurement of cardiac pump function in *ex vivo* hearts perfused in the unloaded, Langendorff mode. We demonstrate that balloons for the device can be manufactured consistently and that their properties are retained over time and with use. Furthermore, as proof of concept, we successfully measured several hemodynamic parameters with our CLD in the isolated, perfused, working (loaded) rat heart. This type of device could be of use in both laboratory experimentation and clinical applications.

The CLD that we describe was sized to fit rat hearts, and is designed for research applications in which characterization of cardiac function is critical. Several research protocols use isolated, perfused hearts in the Langendorff or unloaded mode for cardiac evaluation. This approach, although relatively simple and highly reproducible, has a very limited interest for the assessment of the contractile function, as the LV remains unloaded. Isovolumetric, intra-ventricular balloons are sometimes used with these preparations to monitor LV contractile function. This assessment of LV function is, however, limited to the pressure generated by the LV against a fixed resistance, which is not representative of a clinical scenario. In an *ex vivo* perfused heart, the best current approach to evaluate LV contractile function is the working or loaded configuration, in which physiological flow of perfusate or blood through the heart chambers is respected. The establishment of such a system, however, requires greater technical expertise and is more costly. Our cardiac loading device has the major advantage that it can be simply added to a regular Langendorff (unloaded) heart perfusion system, thereby offering an alternative that enables continuous monitoring of contractile function of the left ventricle during the entire ventricular “loading” and “unloading” phases, without the demands of a traditional working-heart system.

Our results from experiments with isolated, perfused rat hearts indicate that the device is effective for ventricular loading and evaluation of hemodynamic parameters, such as heart rate, developed pressure, dP/dt_max_ and dP/dt_min_. Our findings also indicate that the estimation of the cardiac output is possible. Heart rate measurements look almost identical between those obtained with the CLD and those obtained with the classically loaded heart preparation, as would be expected. However, CLD measurements of developed pressure, dP/dt_max_ and dP/dt_min_ tend to be slightly lower (higher for cardiac output) than those in the classical heart preparation. Importantly, this tendency for lower values is not necessarily problematic; CLD values only need to reflect true heart function. Values measured with the CLD can be adjusted using correction factors for more precise results.

Our cardiac loading device could be of interest for both preclinical and clinical applications. In addition to hemodynamic assessment in isolated, perfused rat hearts, the principle could be applied in larger species, such as rabbits, or pigs. With an adapted design, it could be implanted in an experimental model of heterotopic transplantation[13], thereby permitting characterization of ventricular load (preload and afterload) on the reverse remodeling process[14]. In clinical practice, the CLD principle could be used with an *ex vivo* perfusion apparatus such as the OCS, for the evaluation of graft function before transplantation. This would be of particular interest for DCD heart transplantation since current graft evaluation possibilities remains rudimentary, and may limit the expansion of DCD use in heart transplantation.

We established a ventricle simulator to test our device *in vitro* prior to performing *ex vivo* tests. This *in vitro* system was very valuable for developing our CLD and determining the appropriate estimate for the valve delay time, which is dependent on heart rate. Our results from these *in vitro* tests show that the CLD functions appropriately for at least one hour at a heart rate of 230 bpm and a developed pressure of 100 mmHg.

In order to develop our CLD, it was necessary to establish fabrication techniques for the consistent and reliable production of both VBs and RBs. Importantly, we did not measure any significant difference in PV relationships or maximal working volumes (p>0.05) for VBs among batches. Our results also indicate that the PV relationship for RBs is almost linear, rendering the calculation of elastance by linear regression accurate (R^2^> 0.997). However, we measured a significant decrease in elastance of the RB between batches (p<0.05), when the time between the opening of the silicone bottles and the production of the balloons was longer, as demonstrated by the negative correlation (p<0.05) between the elastance of the reservoir balloons and the 'age' of the silicone. In order to avoid these differences, it is necessary to control and monitor the time between the opening of the silicone bottle and its use. Thus, apart from silicone-age-related differences in elastance for the RB, balloon production is very consistent. Our results from ‘weekly’ and ‘over use’ tests show that the PV properties of both VBs and RBs are stable, with no detectable changes for up to 24 weeks after production, respectively, and no changes after 1 hour use with the CLD (p>0.05).

We have developed a simple, reliable method to manufacture VBs that are very thin, flexible, compliant and with properties that remain constant, and which could be used for other applications, such as isovolumetric, Langendorff heart perfusions. Indeed, in these experiments, a fluid-filled intraventricular balloon, connected to a pressure transducer and inserted into the LV via the mitral valve, is used to measure contractile function (i.e. heart rate, ventricular pressures). Many investigators performing Langendorff heart preparations use standard latex balloons[15–18]; however, these balloons are neither very flexible, nor compliant. Furthermore, latex is cytotoxic, which would limit its adequacy for long-term experimental use. Therefore, investigators often make their own balloons from cling film/plastic wrap[19–21]. However, properties of latex or cling film change over time and with use; therefore, balloons must be changed quite often[22]. Although other methods to produce silicone balloons exist[23], ours is very reproducible, providing balloons with similar volume and shape among batches.

In summary, we have developed a simple CLD and demonstrated that this approach can be used to simulate ventricular loading and monitor hemodynamic function during *ex vivo* perfusion, such as occurs during organ transplantation.

### Limitations

The device we have presented here has been developed for application in rat experimentation and this study should be considered as a proof of concept. Further development and adaptations are required for use in bigger animal species, such as pigs, and before testing in human hearts.

### Outlook

The continuous need to increase cardiac graft availability has led to the expansion of the donor pool, with marginal DBD and DCD and the introduction of *ex vivo* graft perfusion technologies in clinical practice. Now more than ever, there is a need to improve DBD and DCD cardiac graft evaluation, and *ex vivo* graft perfusion permits the development of many potential assessment parameters, including contractile function. However, unloaded perfusions are of limited value and typical working model systems are technically complex in practice. Thus, our CLD could offer a solution for improved assessment of cardiac function prior to transplantation.

Furthermore, many laboratories worldwide are working with *ex vivo*, perfused heart models in a Langendorff (unloaded) mode. Therefore, our device could help to assess cardiac hemodynamic function more accurately. In a next generation, one could also add different physiological and chemical stretchable sensors on the silicone of the VB to improve the methods to evaluate cardiac graft function, which might find clinical applications in a near future.
